# Monoclonal antibodies expression improvement in CHO cells by PiggyBac transposition regarding vectors ratios and design

**DOI:** 10.1371/journal.pone.0179902

**Published:** 2017-06-29

**Authors:** Samira Ahmadi, Fatemeh Davami, Noushin Davoudi, Fatemeh Nematpour, Maryam Ahmadi, Saeedeh Ebadat, Kayhan Azadmanesh, Farzaneh Barkhordari, Fereidoun Mahboudi

**Affiliations:** 1 Biotechnology Research Center, Pasteur Institute of Iran, Tehran, Iran; 2 Medical Biotechnology Department, Semnan University of Medical Sciences, Semnan, Iran; 3 Department of Virology, Pasteur Institute of Iran, Tehran, Iran; Friedrich-Loeffler-Institute, GERMANY

## Abstract

Establishing stable Chinese Hamster Ovary (CHO) cells producing monoclonal antibodies (mAbs) usually pass through the random integration of vectors to the cell genome, which is sensitive to gene silencing. One approach to overcome this issue is to target a highly transcribed region in the genome. Transposons are useful devices to target active parts of genomes, and PiggyBac (PB) transposon can be considered as a good option. In the present study, three PB transposon donor vectors containing both heavy and light chains were constructed, one contained independent expression cassettes while the others utilized either an Internal Ribosome Entry Site (IRES) or 2A element to express mAb. Conventional cell pools were created by transferring donor vectors into the CHO cells, whereas *transposon*-based cells were generated by transfecting the cells with donor vectors with a companion of a transposase-encoding helper vector, with 1:2.5 helper/donor vectors ratio. To evaluate the influence of helper/donor vectors ratio on expression, the second transposon-based cell pools were generated with 1:5 helper/donor ratio. Expression levels in the *transposon*-based cells were two to five -folds more than those created by conventional method except for the IRES-mediated ones, in which the observed difference increased more than 100-fold. The results were dependent on both donor vector design and vectors ratios.

## Introduction

Monoclonal antibodies (mAbs) are among the best-selling class of biopharmaceutics [1]. This rapid growth in demand for mAbs has led to further employ new technologies to increase antibody expression levels in mammalian cells. Chinese Hamster Ovary (CHO) cells are the prominent choice of mammalian expression system [2, 3]. To achieve maximum IgG production in mammalian cells, a balanced expression of both chains is mandatory [4, 5]. Expression of each light and heavy chain- on separate vectors is probably the least effective method to generate a mAb-producing cell line [6]. A rational way to regulate LC: HC ratio is to use a single vector carrying two separate expression cassettes with all required elements [5]: then, both genes will be integrated at the same position but they will be expressed independently [1, 7]. Another approach to express both chains, is to produce bicistronic mRNAs. To obtain bicistronic mRNAs some famous options are included Encephalomyocarditis virus (EMCV), internal ribosome entry site (IRES), and foot-and-mouth disease virus 2A (F2A) peptide sequence elements—[8]. IRES introduces a new ribosome binding site to translate the second gene in a cap-independent manner; however, it leads to lower expression of the second gene [9]. 2A elements have a self-cleavage property; they cleave themselves after translation [10]. Although 2A directs equivalent expression of both genes, it leaves few amino acids at C-terminal region of the first gene. In order to remove extra amino acids, a furin cleavage site can be incorporated into 2A sequences [7, 8].

Conventional gene transfer approaches are based on the random integration of transgenes into the genome of cells; so transgenes expression levels are vastly unforeseeable; therefore, a large number of individual clones should be monitored to find a few high-producing cell lines [11, 12]. Transgenes integration sites and their copy number play major roles in the observed dissimilarity between clonal cell lines [13, 14]. In recent years, different approaches have been employed to obtain constant expression of transgenes [14, 15]. Establishing mammalian cells compatible version of transposons is one of these approaches [16]. Transposable elements such as PiggyBac (PB) have been proved to hold up the integration of transgenes into the genome of mammalian cells [17, 18].

The PB transposon system has one or more transposon donor vectors, which express the transgene(s) and a helper vector encoding the PBase enzyme [19]. PB targets the tetranucleotides TTAA in the genome [20, 21]. Transposition takes place by a "cut and paste" mechanism. PBase identifies the terminal repeats, binds to them, and then excises the whole transposon from its initial place. Afterward, PBase helps the transposon to be inserted to a new chromosomal site [22, 23]. Using PB transposon has some advantages over the conventional transfection methods. Probably, the main advantage is its priority to target the transcriptionally active areas of the genome, which are rich in CpG islands. Furthermore, it would increase transgene integration efficiency [24–26].

In the present study, three different transposon donor vectors were constructed; one carrying two independent mammalian expression cassettes to express both light and heavy chains of an IgG1 molecule,—the others—employing IRES or 2A to produce both MAb- chains under control of a single promoter. Comparisons were performed on different cell pools generated either by conventional gene transfer method or transposition method with different ratios of donor to helper PiggyBac vectors. PB transposition effects were evaluated regarding different vector design and ratios.

## Materials and method

### Vectors construction

**Fig 1:**
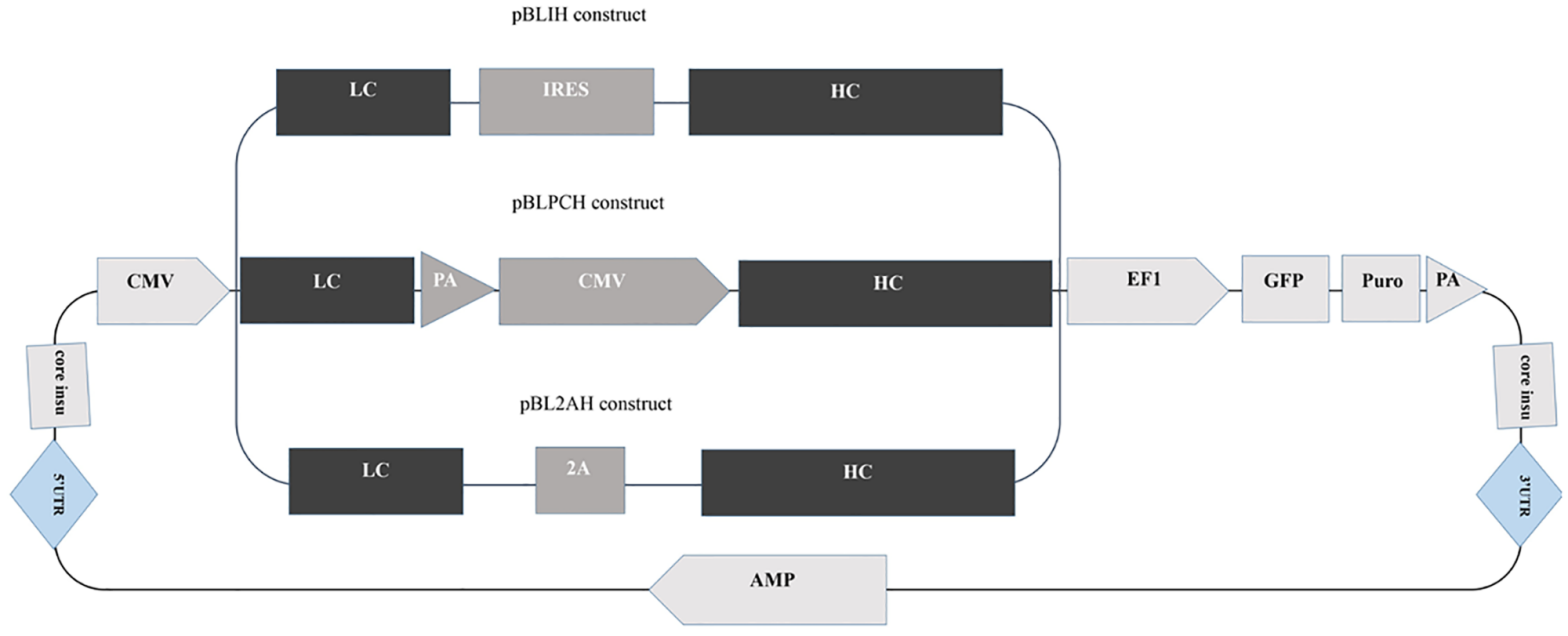
Different donor vectors were constructed. Two independent promoters or one promoter plus IRES or 2A elements control light and heavy chain expression via single donor vectors. The UTRs are the areas, which transposase knows and cuts to transpose the donor vector into the host genome.

The pB513B1 donor vector and pB200A helper vector were purchased (System Bioscience, CA, USA). To construct dual promoter vector, LC and BGH polyA sequences were PCR amplified from the pUC-LC and pTracer-CMV2 (Invitrogen, CA, USA) vectors respectively. After cloning -into an intermediate vector, they were cloned into pB513B1 with the aid of EcoRI/ BamHI enzymes, and pBLP vector was obtained. CMV-HC sequence was sub-cloned from the pTracer-HC vector, into the pBPL vector by BglII/NotI to achieve pBLPCH final construct. LC-IRES-HC and LC-F2A-HC (F2A; furin-containing 2A peptide sequence) fragments were previously constructed in our lab; but, on different conventional vectors. LC-IRES-HC-containing vector was digested with NheI/NotI and the obtained fragment ligated into the pB513B1 to result in pBLIH donor vector. LC-F2A-HC- cloned into pB513B1 with the aid of XbaI/NotI enzymes and pBL2AH were obtained (Fig 1).

### Cell culture

Suspension adopted CHO-S cells were provided (Invitrogen, CA, USA). Cells were maintained in ProCHO5 medium (Lonza AG, Verviers, Belgium). The medium was supplemented with 4mM L-glutamine (Invitrogen, CA, USA), 2mM PenSterp (Invitrogen, CA, USA), and anti-clumping agent (Invitrogen, CA, USA). The cells were kept in a humidified incubator at 37°C with 5% CO2 atmosphere. The cells were cultivated either in T-flasks or shaken in glass bottles. The cells were sub-cultured twice a week at a density of 3×10^5^ cells/ml. Trypan blue exclusion method was used to evaluate cell number and viability.

### Cell transfection

X-tremeGENE HP (Roche, Mannheim, Germany) was utilized to transfect the cells. In Brief, one day before transfection 2 ml ProCHO5 medium without any supplements containing 0.8–1× 10^6^ cells/ml was seeded in 6 well plates. On the day of transfection, 3 μg of DNA and 9 μl of X-tremeGENE HP were diluted in 300 μl of SFM (Invitrogen, CA, USA), respectively. They were incubated at room temperature for half an hour, afterward were added to the cells drop-wise.

### Flow cytometer analysis

As the donor vectors express eGFP, transfection rates in the cells were determined 24 hours post-transfection. A fraction of each cell pool (about 1–2×10^5^ cells) was diluted in PBS and—analyzed with the application of the flow cytometer (CyflowPartec, Görlitz, Germany) with 488 nm excitation and 532 emission wavelengths. After one month selecting the cells with puromycin as well as five months cultivating the cells in a non-selection medium, GFP expression analyses were performed again with the mentioned wavelengths, but only for dual promoter donor vector.

### Generation of stable pools

The cells were transfected in duplicates as described in the previous section. To generate dual promoter-expressing cells, transfections were performed with only pBLPCH vector or with PBLPCH plus pB200-A transposase helper vector with 1:5 or 1:2.5 helper/donor molar ratios. Hence, N-pBLPCH, 1/5-pBLPCH, and 1/2.5-pBLPCH cells were generated respectively. Creation of single ORF mAb expressing cells was performed following a similar procedure. The cells were transfected with only pBLIH or pBL2AH vectors or with these vectors plus PB transposase vector; however, just 1:2.5 helper/donor ratio was employed. The generated cells were named N-pBLIH, 1/2.5-pBLIH, N-pBL2AH, and 1/2.5-pBL2AH with consideration of their donor vector name and their transposase rate. 48 hours post transfection, the media of cells were changed with supplemented ProCHO5 containing 10 μg/ml puromycin (Sigma-Aldrich, Missouri, USA). Every three-four days, the selective medium was replaced for at least one month to obtain stable pools. To evaluate the mAb expression of each stable pool, cells were seeded at the density of 5 × 10^5^ cells/ml and final volumes of 100 ml in shake flasks. Following seven-day batch-mode cultures, cells supernatants were collected to assess their mAb expression.

### Stability study and clonal selection

To assess the stability of mAb expression, the N-pBLPCH, 1/5-pBLPCH, and 1/2.5-pBLPCH cell pools were sub-cultured over five months in a non-selective medium, and mAb expression levels were evaluated every other week. The clonal selection was performed for these three obtained cell pools by means of limiting dilution. In brief, 0.5–1 cell per well was seeded in 96 well plates in 200 μl of supplemented ProCHO5 without puromycin. After 20 days, the cells grown sufficiently were transferred to 24 well plates. After one passage, they were five times diluted in six-well plates, and their supernatants were collected for further analysis after four days.

### Determination of specific productivity and growth rate

To assess the specific productivity of cells, four clones of N-pBLPCH, 1/5-pBLPCH, and 1/2.5-pBLPCH pools were chosen. 2×10^5^ cells of each group were seeded in shaker bottles, and—viable cell density and antibody titer were evaluated whole five days. Viable cell density was determined using trypan blue exclusion method. Doubling time of the cells was calculated by the equation below:

$$Doubling\;time=\frac{duration*\text{log}\left(2\right)}{log\left(Final\;Concentration\right)-log\left(Initial\;Concentration\right)}$$

Specific productivity (Qp) was measured in pg/cell/day using the following equation [2]:

$$Qp=\;\frac{10.\;\;ln\left(\frac{n_{t}}{n_{0}}\right)\Delta P}{\left(n_{t}-n_{0}\right)t}$$

Where *ΔP* is the change in antibody titers (μg/ml) between the first and last days of the evaluation, *n*_*0*_ and *n*_*t*_ are viable cell densities (10^6^ cells/ml) at the beginning and end points, and *t* is culture time in days.

### ELISA assay

Sandwich ELISA was employed to determine the level of mAb expression in cells supernatants. The protocol was as follows: 125 ng/ml of rabbit anti-human gamma-chain specific antibody (Thermo Scientific Pierce, Massachusetts, USA) in Bicarbonate buffer (PH9.4–9.6) was utilized to coat 96 well plates, which were incubated overnight at 4°C afterward. Then, a 0.5% (w/v) BSA-containing PBS buffer was used as the blocking reagent. Diluted cell supernatants were added in the next step, and the captured mAbs were determined by HRP conjugated goat anti-human antibody (Sigma-Aldrich, Missouri, USA) (1/20000 (v/v) diluted in PBS). Eventually, TMB (tetramethyl benzidine) (Sigma-Aldrich, Missouri, USA), the HRP substrate, was added to the plates. After 10 to 20 minutes incubation at the room temperature in the dark, Sulfuric acid (H2SO4) (Merck, New York, USA) 2N was added to stop the reaction, and the plates were read at 450 nm wavelength by ELISA microplate reader (BioTek, Winooski, Vermont, USA). Washing procedures were performed between all steps using PBS buffer containing 0.05% (v/v) tween 20. All incubation times lasted for 1 hour at 37°C; otherwise, it was stated. Human IgG (Genscript, Piscataway, USA) with defined concentration was applied to draw a standard curve.

### Antibody purification

Supernatants of the stable cell pools containing the expressed mAb were collected to be purified using Mab Select column (GE Healthcare, Little Chalfont, UK). Four to five column volume of PBS buffer was used to equilibrate the column. Afterward, the supernatants were loaded. Assessment of the 280 absorbance showed the column loading procedure. Column washing was performed by four to five column volume of PBS buffer. Elution of the attached antibodies occurred with the aid of 0.1 M buffer of sodium citrate, pH3 (Merck, New York, USA). The collected fractions were neutralized with 2M Tris-HCL, pH8 (Merck, New York, USA).

### Sodium dodecyl sulfate-polyacrylamide gel electrophoresis and Western blotting

Purified mAbs were run on the SDS-PAGE gel in both reducing and non-reducing forms. The resulting bands were appeared by means of Coomassie Brilliant Blue staining. For western blotting, 20 μl of each cell supernatant was run on SDS-PAGE gels in the reduced and non-reduced forms, next the bands were transferred to a nitrocellulose membrane using Trans-Blot SD semi-dry transfer cell (Bio-Rad, California, USA). Afterward, the membrane was blocked in 3% (w/v) skim milk for an overnight. HRP conjugated goat anti-human antibody 1:1000 (v/v) diluted was utilized to detect, and 3,3′-diaminobenzidine (DAB; Sigma-Aldrich, Missouri, USA)—used to visualize the bands. Human standard IgG (Genscript, Piscataway, USA) was applied as the positive control in both SDS-PAGE and western blotting. Also as the negative control, 20 μl of the untransfected cells supernatant was run in the western blotting -.

### RNA and DNA extraction

1 × 10^6^ cells in their mid-exponential growth phase were collected, and their total RNA was purified using TRI reagent (Sigma-Aldrich, Missouri, USA) based on the manufacturer's protocol. DNAseI (Fermentas, Thermo Scientific, Massachusetts, USA) treatment was performed to eliminate any possible DNA contaminations. Afterward, cDNA synthesis was done using 400 ng of RNA and Taqman first strand cDNA synthesis kit (Roche, Mannheim, Germany). The same number of the cells with similar conditions was centrifuged, and their DNAs were extracted employing High Pure DNA extraction kit (Roche, Mannheim, Germany). To reveal the concentrations and quality, isolated RNA and DNA samples were assessed with Nanodrop 1000 spectrophotometer (Thermo Scientific, Massachusetts, USA).

### Quantitative real-time PCR

HC and LC mRNA expression levels and their gene copy numbers in CHO cells were determined by means of quantitative real-time PCR (qRT-PCR) using ABI 7500 PCR system (Applied Biosystem, California, USA) and ABI SYBR Green master mix (Applied Biosystem, California, USA). Primer Express 3 software (Applied Biosystems, California, USA) was employed to design HC, LC, GAPDH, and β-actin specific primers (Table 1). Amplifications were done with an initial heat of 95°C for 10 minutes and—followed by 40 cycles of 15s at 95°C and one minute at 60°C. PCR reaction specificity was determined by melting curve analysis. mRNA level evaluations were quantified by a comparative approach using GAPDH as an endogenous reference to normalize the obtained data of amplifications with the Pfaffl method [27]. Gene copy numbers, which were estimated as described previously [2], performed by means of plotting standard curves for the vectors harboring HC and LC genes. β-actin gene was the DNA internal control, and the CHO cells genome size was assumed to be 6.6 pg/cell.

**Table 1:** Sequences of primers used in the real-time PCR reactions.

Gene name	Forward primers	Reverse primers
GAPDH	CACTCTTCCACCTTTGATGCTG	GTCCACCACTCTGTTGCTGTAGC
β-actin	GAAGTGTGACGTCGACATCCGCAAAGAC	GGTTGACCTGGAAGGGCCCATCATG
Heavy chain	CGACGGCTCCACAAACTATAATCC	TGCCAGTGACCGAAATAGTGAGAC
Light chain	CAGAGTGTGGACTACGATGGAGAC	CGGAGCCTGAGAACCTGGATG

### Statistical analysis

Obtained data was statistically analyzed in GraphPad Prism 6 software using the one-way ANOVA test to determine whether differences in mAb expression of generated cells pools were significant. To this end, a value of P<0.05 was set for analyses as the level of statistical significance. That is to say, all cell pools were generated in duplicates and ELISA and real-time assays were performed as technical triplicates.

## Results

### The impact of different ratios of PB transposase on generation of stable pools

**Fig 2:**
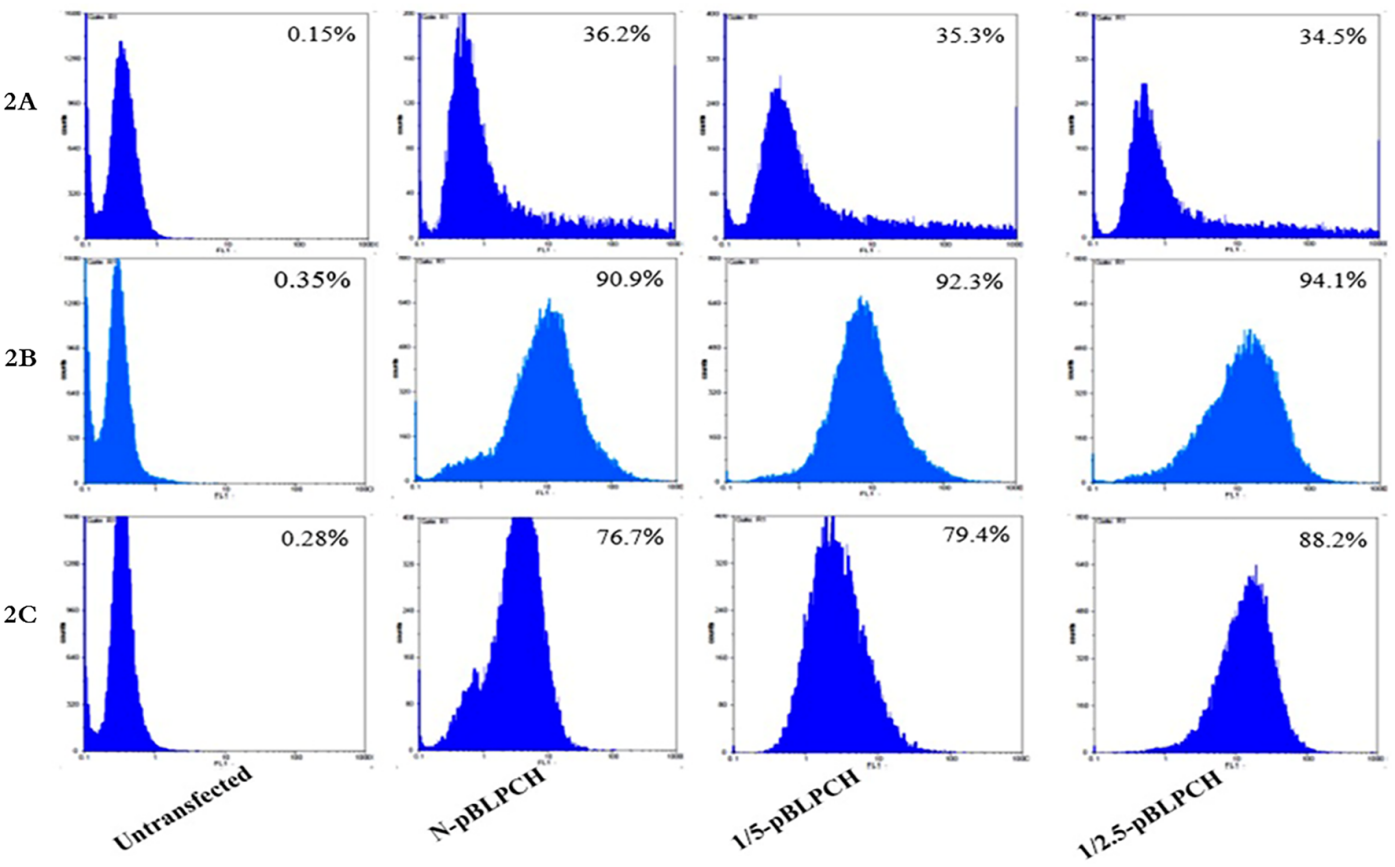
Flowcytometer analysis of created cell pools. The pBLPCH vector contained GFP gene- used as the donor vector -, it was excited at 488 nm wavelength and—emitted at 532 nm. **A**, 24 hours post transfection. Transfection rates of all the three cell pools were 33–35%. **B**, After one month of selection with 10 μg/ml puromycin. Most of the cell populations had expressed GFP protein. **C**, six months post transfection and five months in non-selection medium the 1/2.5-pBLPCH cells retained GFP expression in more quantity than the other cell pools. Untransfected; The untransfected cells as the negative control, N-pBLPCH cells; created by pBLPCH only, 1/5-pBLPCH cells; created with 1:5 ratio of transposase/transposon (pBLPCH) vectors, and 1/2.5-pBLPCH cells; created with 1:2.5 ratio of transposase/transposon vectors.

**Fig 3:**
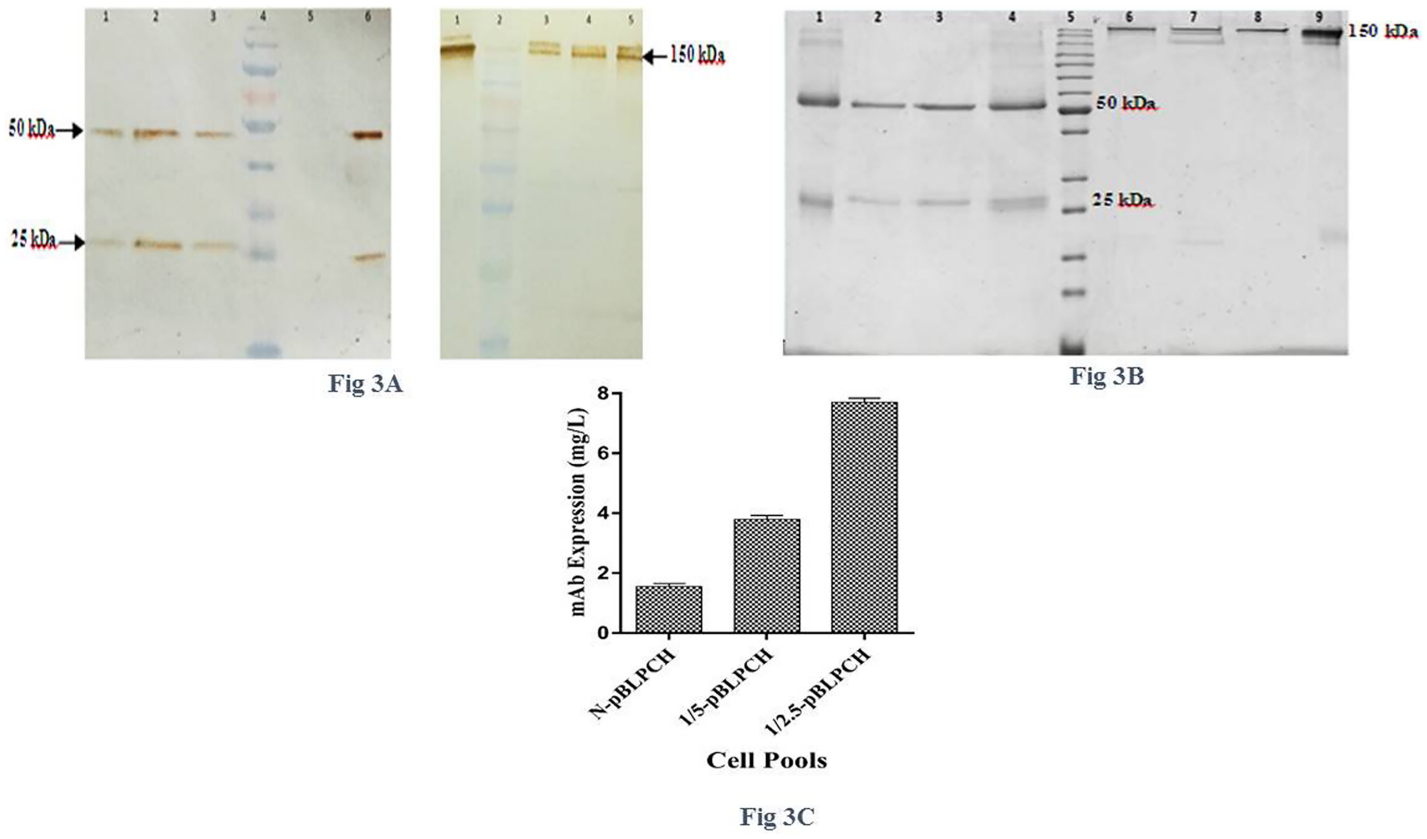
Generation and evaluation of dual ORF vector containing cells. Three different cell pools were created, One by transfection of the pBLPCH vector only as a donor vector in the N-pBLPCH cells and the others by transfection of transposase and pBLPCH vectors with 1/5 and 1/2.5 ratios of helper to donor to produce 1/5-pBLPCH and 1/2.5-pBLPCH cells respectively. **A**, Western-blot analysis of expressed mAb in CHO cell pools supernatants in reduced and non-reduced forms. Goat anti-human HRP conjugated antibody was used as a detector. In the reduced form (left figure) Lane 1, N-pBLPCH cells, lane 2, 1/2.5-pBLPCH cells, lane3, 1/5-pBLPCH cells, Lane 4, pre-stained protein marker (10–170 kDa), lane 5; -untransfected cells supernatant (negative control), and lane 6; purified human IgG as a positive control, in the non-reduced western-blotting (right figure); lane 1,positive control, lane 2, protein marker, lane 3, N-pBLPCH, lane 4, 1/5-pBLPCH and lane 5, 1/2.5-pBLPCH. **B**, SDS-PAGE analysis. Protein-A purified mAbs were run in both reduced (lanes 1–4) and non-reduced (lanes 6–9) forms. Lanes 1 and 9; purified human IgG (positive controls), lanes 2 and 8; N-pBLPCH cells, lanes 3 and 7; 1/5-pBLPCH cells, lanes 4 and 6; 1/2.5-pBLPCH cells, and lane 5; protein marker (10–200 kDa). **C**, Cell pools antibody titers were measured by means of ELISA. Error bars indicate SD of triplicate measurements. ANOVA was used to detect statistically significant differences between the generated pools (P< 0.05).

To evaluate the impact of PiggyBac transposase/transposon ratio on the generation of CHO stable pools containing dual promoter vector, three groups including the N-pBLPCH. 1/5-pBLPCH and 1/2.5-pBLPCH cells were defined. As the manufacturer of PB transposon vectors recommended to transfect the cells with helper/donor ratio of 1:5 or 1:2.5 to get the best results, the present researchers assessed both ratios on mAb expression to specify the best one. Flow cytometry assay indicated that transfection rates were about 33%-36%, regardless of the existence of transposase vector (Fig 2A). The cells were kept for one month in the selection media (Fig 2B). To demonstrate the expressed mAbs in protein level, SDS-PAGE and Western blotting were performed. In the reduced forms, light chains with 25 kDa and heavy chains with 50 kDa bands were observed while in the non-reduced ones, whole antibodies were stopped at the top of the SDS-PAGE gel. (Fig 3A and 3B). Expression quantification by ELISA showed that the 1/2.5-pBLPCH cells had the most expression level, 7.7 mg/l, which was about five times more than—the N-pBLPCH cells with 1.55 mg/l. Moreover, the expression of the 1/5-pBLPCH cells was 3.8 mg/l, just 2.4 folds more than—the control N-pBLPCH cells (Fig 3C).

### PiggyBac transposition effect on single-ORF donor vectors containing cell pools

**Fig 4:**
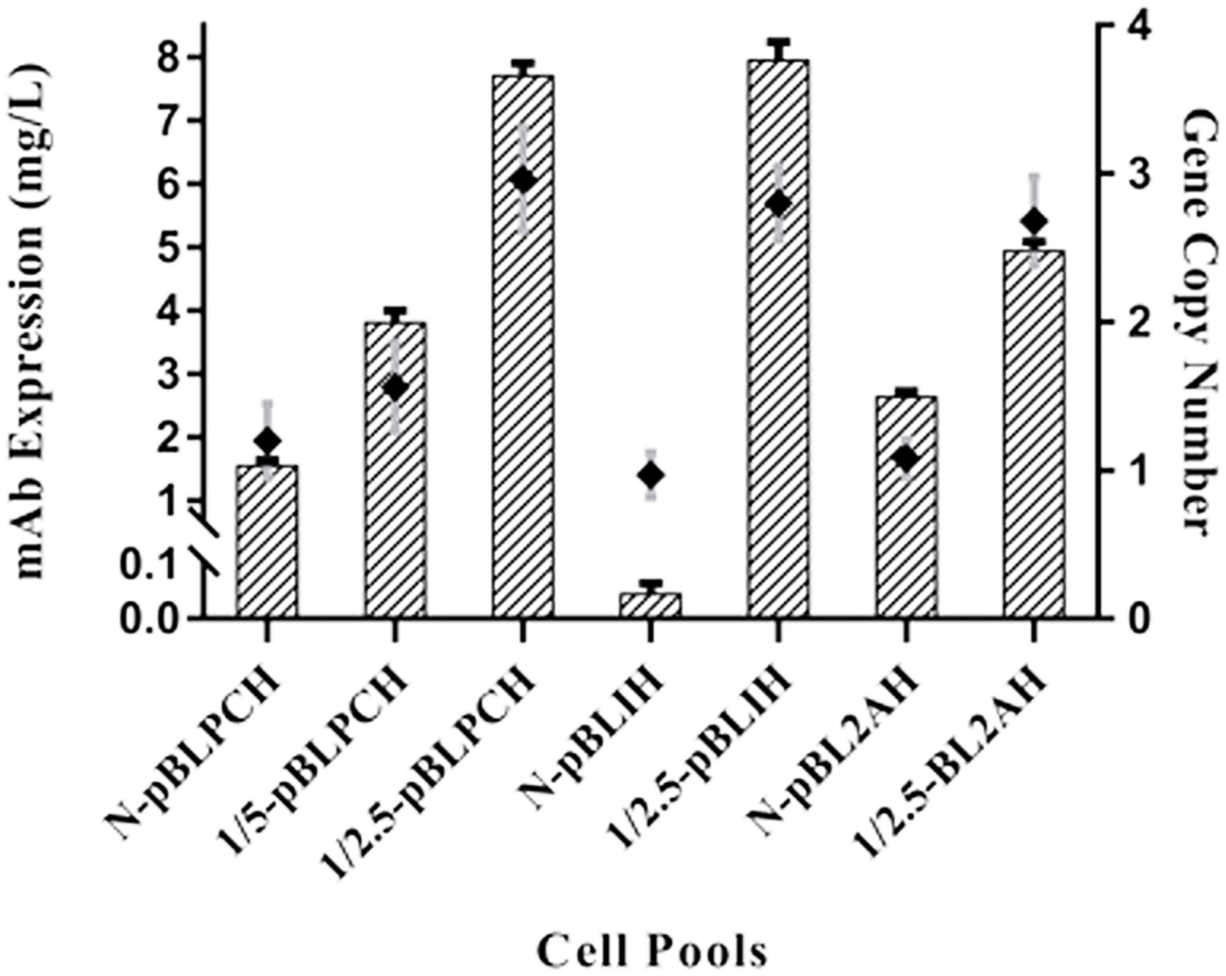
Expression evaluations of created pools by ELISA assay. Expression levels of all the created pools evaluated by ELISA assay while quantitative real-time PCR was employed to determine gene copy numbers. Non-transposed cells had similar gene copy number although N-pBLIH cells had—low expression level. Transposition based cells had similar copy numbers as well, however, their expression levels were also more similar. Error bars indicate SD of triplicate measurements. ANOVA was used to detect statistically significant differences between the generated pools (P< 0.05).

To assess PB transposition effect on single ORF donor-containing cell pools, four cell pools were generated. N-pBLIH and 1/2.5-pBLIH were IRES-containing pools; while N-pBL2AH and 1/2.5-pBL2AH were 2A involved ones. ELISA analysis of—these cells supernatants showed that transposition was effective to increase mAb expression levels. In the 2A containing pools, this effect increased expression level two-fold; N-pBL2AH cells produced 2.6 mg/l antibody while 4.93 mg/l was the expressed antibody titers of 1/2.5-pBL2AH cells (Fig 4). In comparison with all the other cell pools, N-pBLIH had the lowest expression level with less than 50 μg/l expression quantity. Despite poor expression amount of IRES-containing non-transposed cells, the expression level of the 1/2.5-pBLIH cell pools was as high as 1/2.5-pBLPCH, they produced 7.9 mg/l (Fig 4).

### Western blotting analysis of single-ORF expressing cells

**Fig 5:**
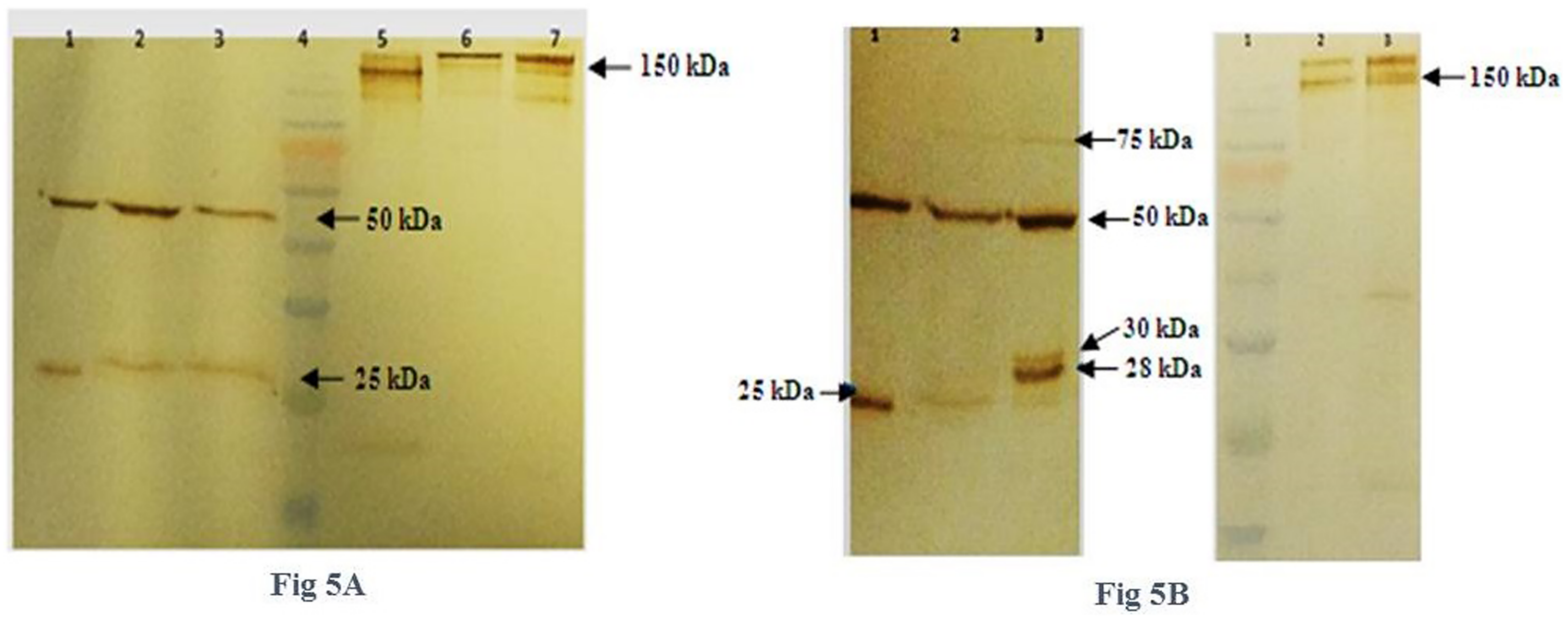
Western-blot analysis of single ORF donor vectors containing cell pools. IRES and 2A elements were used to express light and heavy chains in bicistronic mRNAs. **A**, Western-blotting of IRES-containing cells in reduced and non-reduced forms. Lanes 1–3 are reduced and lanes 5–7 are non-reduced. Lane 1 and 7, purified IgG as a positive control, lane 2 and 6 purified mAb produced by N-pBLIH cells (due to their low expression profile western-blot had done on their purified product), lane 3and 5, the supernatant of 1/2.5-pBLIH cell pools, lane 4, pre-stained protein marker (10–170 kDa). **B**, Western-blot analysis of 2A harboring cells, left figure in reduced and right figure in non-reduced forms. In reduced picture, lane 1, positive control, lane 2, N-pBL2AH cells supernatant, lane 3, 1/2.5-pBL2AH cells supernatant, 25, 28 and 30 kDa light chains appeared in N-pBL2AH and 1/2.5-pBL2AH lanes respectively. Furin peptidase didn't work in the latter. In the non-reduced picture, lane 1, pre-stained protein marker (10–170 kDa), lane 2, N-pBL2AH and lane 3, 1/2.5-pBL2AH cells supernatants.

Western blot analyses were done in both reduced and non-reduced forms for 2A and IRES harboring cells on their supernatants. As it is demonstrated in Fig 5A, both IRES-bearing cells produced mAbs with a typical LC and HC band size, similar to dual promoter cells and the positive control. N-pBLIH had a low expression profile; hence, its western blotting was done after purification. In 2A-containing mAbs, LC bands had different sizes (Fig 5B). In N-pBL2AH cells, there was a dominant 25 kDa light chain band with a very faint band on 30 kDa areas, which revealed—both 2A and furin elements had worked well. However, in 1/2.5-pBL2AH, there were three bands including 25, 28, and 30 kDa. A faint 75 kDa band appeared in both cell pools correspond to the LC-2A-HC fusion protein. In the non-reduced blots, all four mentioned cell pools contained some aggregates similar to those of the positive controls.

### Real-time quantifications, gene copy number, and mRNA fold-induction analysis

**Fig 6:**
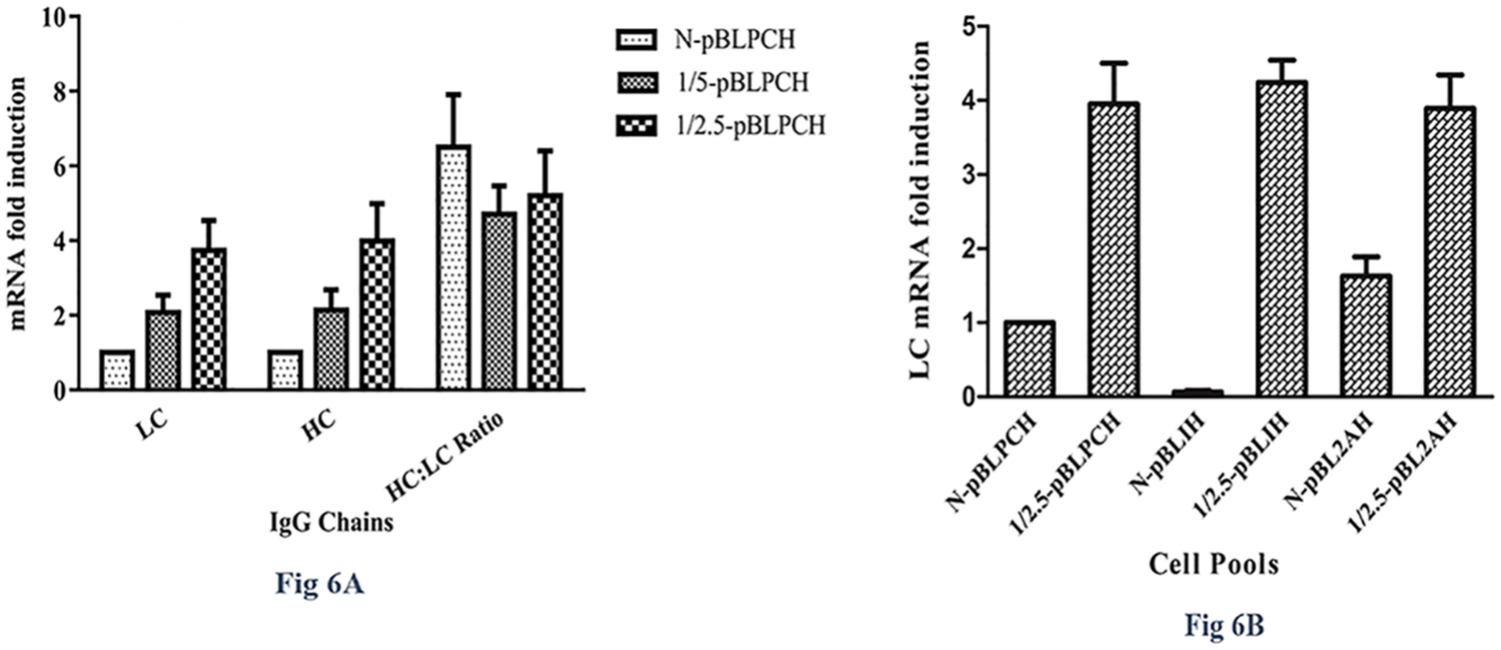
mRNA levels in established pools were assessed by quantitative real-time PCR. **A**, In dual promoter bearing cells both light and heavy chains, are expressed via an independent expression cassette. Transposition effect was assessed at the level of transcription of each chain and as it is demonstrated in the picture both chains expression rate increase in a similar way. HC to LC ratio analysis showed that HC in all dual promoter cells was produced in a greater amount. **B**, Light chain mRNA levels in single ORF mAb expressing cells were evaluated by real-time PCR using N-pBLPCH-LC mRNA as the control gene. Error bars indicate SD of triplicate measurements. ANOVA was used to detect statistically significant differences between the generated pools (P< 0.05).

In dual-promoter mAb-expressing cells, both HC and LC mRNA levels were measured by the already described qRT-PCR. HC and LC levels changed similarly in the conventional versus transpositional cell pools. LC fold-induction rates were 1, 2.07, and 3.74 in the N-pBLPCH, 1/5-pBLPCH, and 1/2.5-pBLPCH cells, respectively; while HC levels were 1, 2.14, and 3.99 in the mentioned cells (Fig 6A). As LC and HC were expressed by two independent promoters, their expressions were expected to be different. Hence, as PCR efficiency for both HC and LC genes were almost equal, we compared their levels of expression. LC played the role of the control gene, and HC gene was considered as the target one. The obtained results had been demonstrated that HC was expressed in higher amounts in all the three different cells, by 6.2, 4.7, and 5.3 fold changes in N-pBLPCH, 1/5-pBLPCH, and 1/2.5-pBLPCH cells, respectively (Fig 6A). Similarly, qRT-PCR reactions were performed to quantify LC mRNA levels in single-ORF expressing cells. With the application of LC mRNA level of N-pBLPCH cells as the control gene, mRNA levels of the other cells were compared with it. In the N-pBL2AH and 1/2.5-pBL2AH cells, fold-inductions were observed 1.63 and 3.89 times, respectively. On the other hand, 0.06 and 4.2 times fold-changes were detected in N-pBLIH and 1/2.5pBLIH cells, respectively (Fig 6B).

To determine gene copy number, qRT-PCR was carried out on extracted genomic DNA of the cells with HC primers. All three non-transposed cells had almost one copy of the donor vector; in fact, 1.2 copies in N-pBLPCH, 0.97 copies in N-pBLIH, and 1.08 copies in N-pBL2AH cells were detected. N-pBLPCH and 1/5-pBLPCH cells revealed almost similar gene copy number, and both of them had more than one copy. In contrast, Higher copy numbers existed in 1/2.5 transposon-based cells. These cells had about three copies of the HC gene; 2.96, 2.80, and 2.68 were detected in 1/2.5-pBLPCH, 1/2.5-pBLIH, and 1/2.5-pBL2AH, respectively (Fig 4).

### The effect of transposition on long-term expression stability

**Fig 7:**
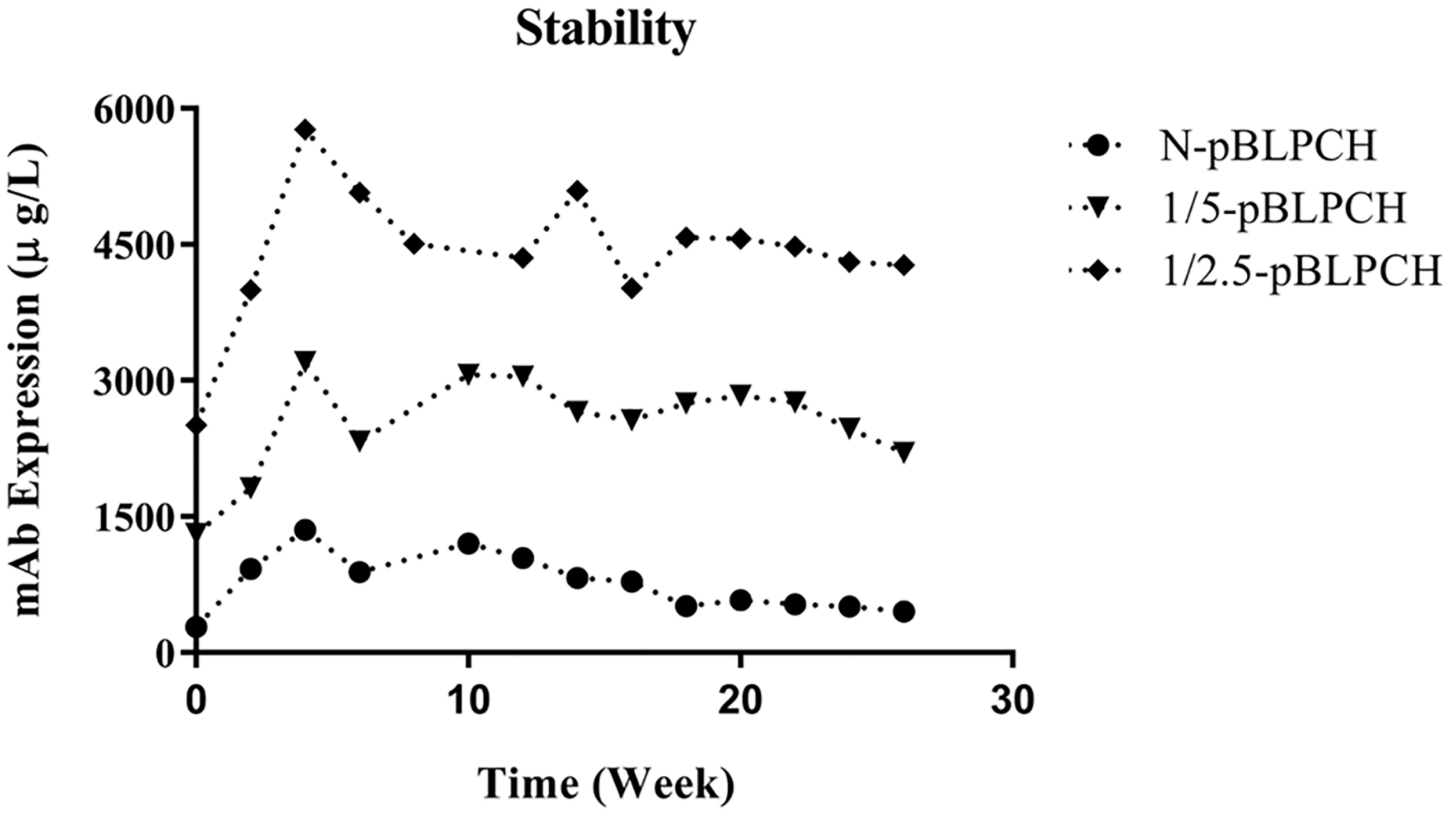
Stability study of dual promoter created pools. Dual promoter cell pools were cultured about six months and their levels of expression were evaluated every other week by the aid of ELISA. As it is displayed in the picture transposition based cells maintained their superiority over the evaluation period but conventional created cells started losing their productivity after 10 weeks. N-pBLPCH cells; created by pBLPCH only, 1/5-pBLPCH cells; created with 1:5 ratio of transposase/transposon (pBLPCH) vectors, and 1/2.5-pBLPCH cells; created with 1:2.5 ratio of transposase/transposon vectors.

After maintaining the pools without selection pressure for five months, flow cytometry analysis of GFP-expressing cells showed that 1/2.5-pBLPCH cells had the most fluorescent signal while 1/5-pBLPCH and N-pBLPCH cells revealed almost 10% less fluorescent signal. Although the percentage of positive cells in the 1/5-pBLPCH and N-pBLPCH cells were almost identical, the fluorescent intensity in the 1/5-pBLPCH cells was more (Fig 2C). In the stability study, the PB transposase harboring cells retained their superiority. During the experiment, 1/2.5-pBLPCH cells had the highest level of expression with more than four-fold and two-fold expressions in comparison with both N-pBLPCH and 1/5-pBLPCH cells, respectively (Fig 7). The N-pBLPCH cells started losing their expression from week 10, and by week 26 they had lost more than 50% of their expression level; while antibody expression of the transposon-based cells was almost stable over time. 1/5-pBLPCH and 1/2.5-pBLPCH lost 25%, and 10% of their expression level, respectively.

### PiggyBac transposition effect on clonal cell line generation and specific productivities of cells

**Fig 8:**
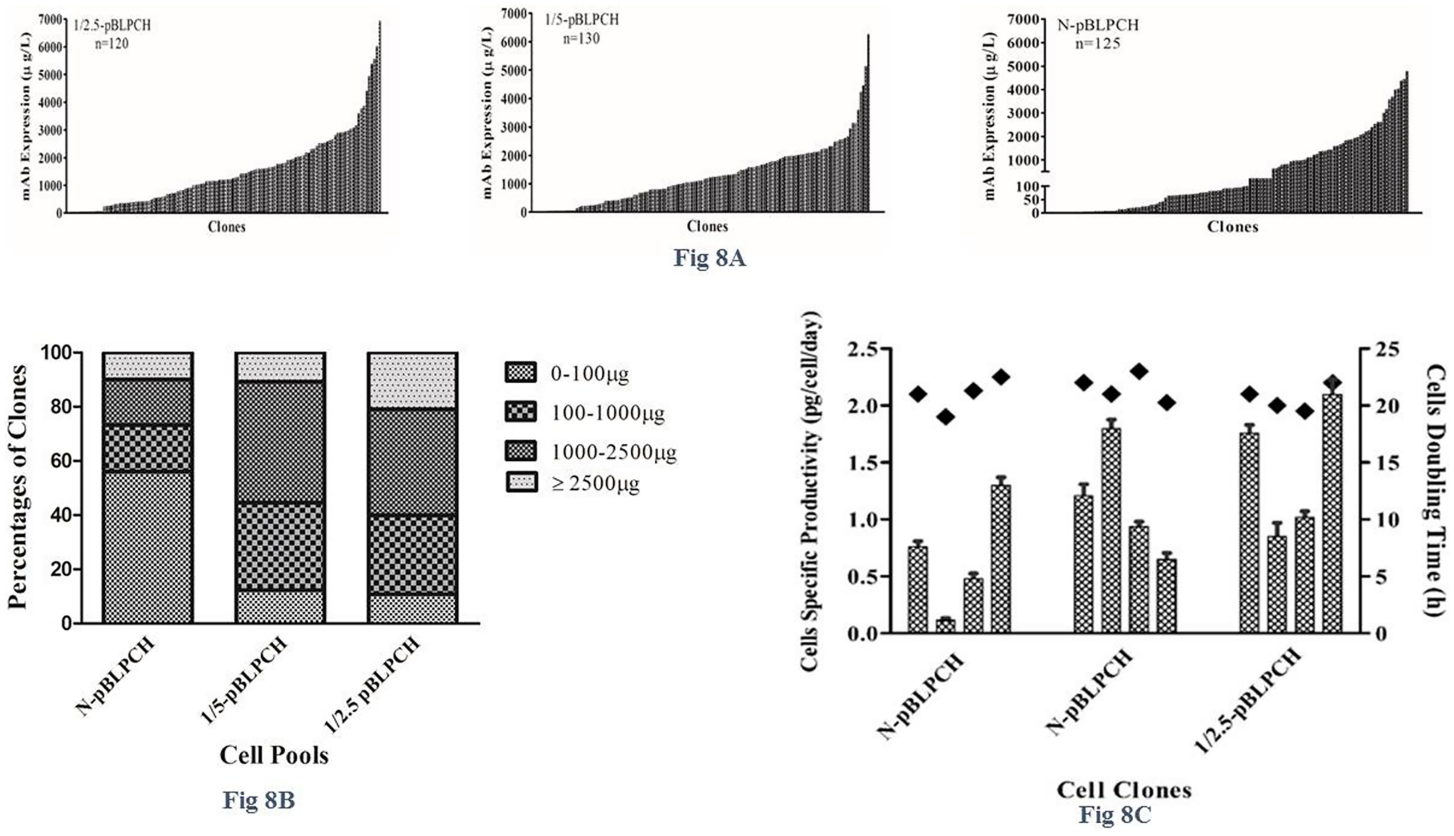
mAb expression assessment in the clonal cell lines. Dual promoter cell pools underwent clonal selection using limiting dilution method. **A**, In1/2.5-pBLPCH cells, 120 clones, in 1/5-pBLPCH cells, 130 clones, and in N-pBLPCH cells, 125 clones were recovered. Transposition based cells showed more productivity in comparison to conventional ones. **B**, More than 60% of clones in 1/2.5-pBLPCH cells had more than 1000μg/L expression while a similar percent of N-pBLPCH derivative cells had less than 100μg/L expression level. Around 21% of clones derived from 1/2.5-pBLPCH parental cells produced mAb more than 2500μg/L whereas two other groups had a comparable number of clones in this area and more than 10% of their clones expressed mAb in this level. **C**, Four clones of each pool were assessed for specific productivity and growth rates. As it is demonstrated, specific productivities of conventional clones were the lowest and transposition based clones were more productive. Clones growth rate were comparable in all evaluated clones. Error bars indicated SD of triplicate measurements.

120, 130, and 125 clones were recovered from 1/2.5-pBLPCH, 1/5-pBLPCH, and N-pBLPCH cell pools, respectively (Fig 8A). In 1/2.5-pBLPCH cells, 21% of clones expressed more than 2500 μg of mAb; however, 10.8% and 10% of clones expressed more than 2500 μg in 1/5-pBLPCH and N-pBLPCH cells, respectively. In N-pBLPCH cells, 56% of clones expressed less than 100 μg; while the observed values were 11% and 12.3% in 1/2.5-pBLPCH and 1/5-pBLPCH cells, respectively (Fig 8B). Four clones with different expression profiles of each pool were chosen to address specific productivity and growth rates. Likewise, clones of N-pBLPCH cells, had lower specific productivity in comparison with those of 1/5-pBLPCH and 1/2.5-pBLPCH cells, with the latter presenting the maximum productive clones (Fig 8C). -, Growth rates of all cells were 20–23 hours, regardless of transposase activity (Fig 8CS).

## Discussion

In the present study, three different donor vectors were constructed, which are able to encode both light and heavy chains of a monoclonal antibody. One vector expressed the mAb through two independent expression cassettes while the other ones produced bicistronic mRNAs either with the aid of IRES or 2A elements. At first, the dual promoter-containing cells were generated. Two pools were constructed using PiggyBac transposon system, the applied helper/donor ratios were 1:5 and 1:2.5 in the 1/5-pBLPCH and 1/2.5-pBLPCH cells, respectively. The third pool was created with donor vector only, named N-pBLPCH, to be compared with transposition ones. The obtained data had been demonstrated that transposon-based dual promoter cells, had superior production over the N-pBLPCH cells. In the current study, 1:2.5 and 1:5 ratios did not cause similar effects on mAb production although they both increased the expression; 1:2.5 had a greater impact. In a previous study conducted by Matasci et al., 2011, in which PB transposon was utilized to express TNFR:FC, there had been no considerable difference in the expression amounts with different helper/donor ratios; while in the present study the ratios had an important effect. Similar results were obtained from the real-time quantitative mRNA expression evaluation, which approved gene expression had been enhanced at the transcription level.

In the IRES-containing cells, N-pBLIH conventionally-made cells had—low expression profile although the 1/2.5-pBLIH cells had much more expression level, which was equivalent to the 1/2.5-pBLPCH cells. Not only N-pBLIH cells expressed antibody far less than their transposon-based cells, but also their expression levels were negligible in comparison with the other conventionally-created cells, N-pBLPCH and N-pBL2AH, even though a wild-type EMCV-IRES sequence had been incorporated into the donor vector [28]. Moreover, in a parallel study performed in our lab with another enzymatic targeting approach (PhiC31 integrase) using IRES to express a mAb, similar outcomes were observed [29].

The N-pBL2AH cells, which utilized 2A element, had the highest expression level among the conventionally-made cells. In comparison with the N-pBLPCH cells, 2A-harboring cells had 1.6 times more expression titer. This superiority may be due to the more balanced expression of the both chains. Coupled with mRNA fold-induction analysis, it had more light chains expressed with an equal amount of heavy chains while in dual promoter mAb expressing cells, heavy chains was transcript far more than the light chains. The more transcription rate of the heavy chain in dual promoter cells may be due to a longer CMV promoter sequence which is applied to encode it. The second promoter which is cloned into the vector is two times longer than vector's own CMV promoter which expresses the light chain.

To remove extra amino acids that remain following 2A cleavage, furin recognition site was incorporated at its upstream. As it had been demonstrated in the previous studies, furin or 2A elements might not work properly all the time. If they failed to efficiently cleave, light or heavy chain bands with different molecular weights would be detected in the western blot analysis [1, 8, 30]. Likewise, Western blot analysis of the 1/2.5-pBL2AH cells' supernatant illustrated that two extra bands of the light chains were around 30 kDa. The smaller one, 28 kDa band, might represent that 2A had worked but furin had not [8]. The 30 kDa band might contain HC signal peptide, which means neither 2A nor furin had worked, and cleavage of the heavy chain signal peptide had caused the light chain to be separated [30]. If none of them worked, a 75 kDa band would appear on the membrane, which corresponds to a fusion protein (LC-F2A-HC) as a faint 75 band was observed in 2A harboring cells.

Notably, the light chain abnormal bands were dominant in transposed 2A-bearing cells (1/2.5-pBL2AH), whereas conventionally-made 2A cells consisted of a main normal 25kDa light chain. Probably, higher expression in transposed cells caused furin and/or 2A not -able to work properly, as some studies suggested to overexpress furin in mAb-expressing cells to solve the problem [31, 32]. That is to say, it is assumed that lower expression of the 1/2.5-pBL2AH cells in comparison with the other two 1:2.5 helper to donor ratio transposed cells might result from the malfunction of furin/2A in the mentioned cells. However, further experiments should be performed to make accurate and strong conclusions.

Previous studies suggested that PBase transposed the transgene in transcriptionally active areas of the genome so the mAb would be expressed more efficiently; and/or more gene copies might be integrated into the genome, also can increase the gene expression [33]. The results of the present study had been demonstrated that both justifications are plausible. The 1/5-pBLPCH cells expressed antibody 2.4 fold more than N-pBLPCH cells despite the fact that both cells had identical gene copy numbers. However, in the 1/2.5pBLPCH cells with five-fold more expression rate than the conventional cells, it also had more gene copy number. It can be proposed that more expression stability which was observed in transposon-based cells might be due to the site of genomic integration and/or gene copy number. Although gene targeting approaches are not as effective as gene amplification methods [34], but, in a study, employing advantages of PB transposon combined with the GS amplification procedure has led to a significant improvement in mAb expression in CHO cells [35]. It demonstrates that transgene amplification in active areas of the genome would result in extraordinary levels of expression.

All three dual promoter cell pools went through limiting dilution process to obtain single clones. Expression homogeneity was observed much more in the transposon-based clones. Above all, the1/2.5-pBLPCH cells exhibited its highest level of expression over both 1/5-pBLPCH and N-pBLPCH cells in clone generation along with its significant impact on the creation of cell pools. In particular, clonal selection data supported expression outcomes of the cell pools: although, lower numbers in the cell counts and product durations led to lower expression in comparison with the cell pools. Greater numbers of clones should be monitored to validate these findings.

To conclude, the results of the current study illustrated that not only PB transposition is a valuable tool to generate mAb-expressing cells over conventional method, but the helper to donor vector ratios has an important effect to achieve better outcomes. All things considered, donor vector properties have great impacts on mAb expression in CHO cells. Utilization of two independent expression cassettes has superiority over single ORF constructs bearing IRES or 2A elements; the latter disrupts the quality of expressed mAb by incompetent cleavages; while conventionally-made IRES-mediated cells lead to inadequate expression level. Not to mention, more detailed investigations are required to be conducted to shed more light on vector design and PB transposition influences on cell line development to attain better consequences.
